# New Insights on the Male and Female Reproductive Organs of *Centrorhynchus globocaudatus* (Acanthocephala), Intestinal Parasite of Birds of Prey

**DOI:** 10.3390/cells13040356

**Published:** 2024-02-18

**Authors:** Bahram Sayyaf Dezfuli, Flavio Pironi, Emanuele Rossetti, Holger Herlyn

**Affiliations:** 1 Department of Life Sciences & Biotechnology, University of Ferrara, St. Borsari 46, 44121 Ferrara, Italy; 2 Institute of Organismic and Molecular Evolution, Anthropology, Johannes Gutenberg University Mainz, 55122 Mainz, Germany

**Keywords:** acanthocephalan, ovarian ball, ultrastructure, uterine bell, male Saefftigen’s pouch

## Abstract

Acanthocephalans are dioecious parasites that gain sexual maturity in the alimentary canal of their definitive hosts (gnathostome vertebrates). This initial survey by light and transmission electron microscopy was conducted on the functional organization of the ovarian balls and uterine bell in mature females and on Saefftigen’s pouch and the copulatory bursa in males. We studied these structures via the example of *Centrorhynchus globocaudatus* (Palaeacanthocephala) in *Falco tinnunculus* and *Buteo buteo*, from the Province of Ferrara (Northern Italy). Our study confirms that the ovarian balls have surface microvilli and consist of a multinucleate supporting syncytium and a cellular region with oogonial syncytium, single germ cells, zygotes, and shelled eggs. Germ cells are embedded in the supporting syncytium. The ultrastructural features of these components and data on fertilization, shell formation, and release from the ovarian ball, alongside insights into the likely egg sorting function of the uterine bell, are provided. We also present light and electron microscopy observations of Saefftigen’s pouch and a suggestion regarding its hydrostatic functioning in the eversion of the copulatory bursa.

## 1. Introduction

Acanthocephala (thorny-headed worm) is a group of endoparasitic helminths with about 1200 described species [1]. These dioecious parasites [2] are readily recognizable by their mostly reversible proboscis attachment organ armed with hooks at the anterior body pole. They occur worldwide, use mandibulate arthropods as intermediate hosts, and reach sexual maturity in the alimentary tract in a wide phylogenetic range of gnathostome vertebrates, especially actinopterygian fishes and tetrapods [3,4].

In female development, the primordial gonads are supposed to give rise to a large number of ovarian balls that float in the fluid-filled trunk or metasoma. In fact, ovarian multiplication [5,6] is one of the most striking peculiarities in the development and maturation of female acanthocephalans. The high productivity of the fragmented ovaries or ovarian balls is reflected in the enormous amounts of embryonated eggs released by females upon insemination. Based on experimental infections, estimates range from 2000 shed eggs per day in the case of *Polymorphus minutus* parasitizing domestic ducks [7] to 260,000 eggs in daily production for *Macracanthorhynchus hirudinaceus* infecting pigs [8]. Nevertheless, only a few scattered surveys have been performed on the structure and cytology of acanthocephalan ovarian balls. Early observations of the histology of the female germ cells occurred more than 100 years ago [9,10,11]. Subsequent studies mentioned that the ovarian balls consist of two regions, a multinucleate syncytium and a cellular zone, and microvillous protrusions cover the ovarian ball surface [12,13]. In addition, the ovarian ball tissue in the immature stages of three species of Palaeacanthocephala, namely young *Corynosoma semerme*, *C. strumosum* from a fish paratenic host, and the cystacanth stage of *Echinorhynchus gadi* from an amphipod intermediate host, was detailed in Peura et al. [14], and Schmidt [15] provided a scheme of an ovarian ball in a palaeacanthocephalan cystacanth.

The metasomal body cavity includes a so-called ligament sac that posteriorly connects with the efferent system for mature eggs, comprising the uterine bell, uterus, and vagina [2,16,17]. The uterine bell forms the inner opening of the female efferent duct [18]. It is likely that the uterine bell allows mature eggs to pass into the uterus while withholding immature eggs, thus functioning as an egg sorting organ [16,18,19].

In the male reproductive system, the prime example of an organ with unknown function is the muscular Saefftigen’s pouch. Together with the retractable campanulate bursa and the penis, Saefftigen’s pouch is regarded as part of the male copulatory structure. However, only a few hand drawings depict this organ in larvae [15] and adult worms [20,21,22], and it remains unclear whether Saefftigen’s pouch actually participates in the extrusion of the bursa, as suggested by the juxtaposition of both differentiations [23].

The present study is intended to expand our knowledge on the morphology and ultrastructure of the ovarian balls and the uterine bell in female and Saefftigen’s pouch and bursa in male acanthocephalans. Additional data are provided on the male cement gland and vesicula seminalis. We focus on a palaeacanthocephalan, *Centrorhynchus globocaudatus*. Using light and transmission electron microscopy, the present survey covers the functional organization of the ovarian balls and the uterine bell in mature, inseminated female *C. globocaudatus*, and Saefftigen’s pouch and the copulatory bursa in males.

## 2. Materials and Methods

### 2.1. Birds and Sampling

During 2022, on three occasions, ten moribund or dead birds of prey *Falco tinnunculus* and *Buteo buteo* were collected by hunting guards during their surveillance inspection of the rural area of Ferrara (Northern Italy). Carcasses were carried to the Experimental Zoo-Prophylactic Institute of Ferrara for routine monitoring for public health in search of West Nile Virus or New Castle Disease, both of which use birds of prey as a vector. After the dissection of the animals, the digestive tract was removed from each animal and fixed in 10% neutral buffered formalin and sent within 24 h to the Department of Life Sciences and Biotechnology of the University of Ferrara, to be examined for enteric helminths.

### 2.2. Light and Transmission Electron Microscopy (TEM)

Numerous specimens of *C. globocaudatus* (Zeder, 1800) Lühe, 1911 (Palaeacanthocephala: Centrorhynchidae) were isolated from the intestines of these birds of prey. For histological examination, 15 males and 20 females (Figure S1) were dehydrated through an alcohol series and then paraffin-wax-embedded using a Shandon Citadel 2000 tissue processor. Multiple 7 μm sections were taken from each tissue block, stained with Alcian Blue, Hematoxylin and Eosin, and/or Giemsa, and examined and photographed using a Nikon Microscope ECLIPSE 80i (Tokyo, Japan). For TEM analysis, 20 mature female and 15 male acanthocephalans were transferred into 2.5% glutaraldehyde in 0.1 M cacodylate buffer, pH 7.2, post-fixed in 1% osmium tetroxide in the same buffer for 1.5 h, dehydrated in a graded acetone series, and embedded in epoxy resin. Semi-thin sections of the embedded samples were cut on a Reichert Om2 ultramicrotome with glass knives, stained with toluidine blue, and examined and photographed using the above instrument. Sections were obtained, stained, and observed with a Talos L120C transmission electron microscope (MA, USA). 

## 3. Results

### 3.1. Light Microscopy

**Figure 1:**
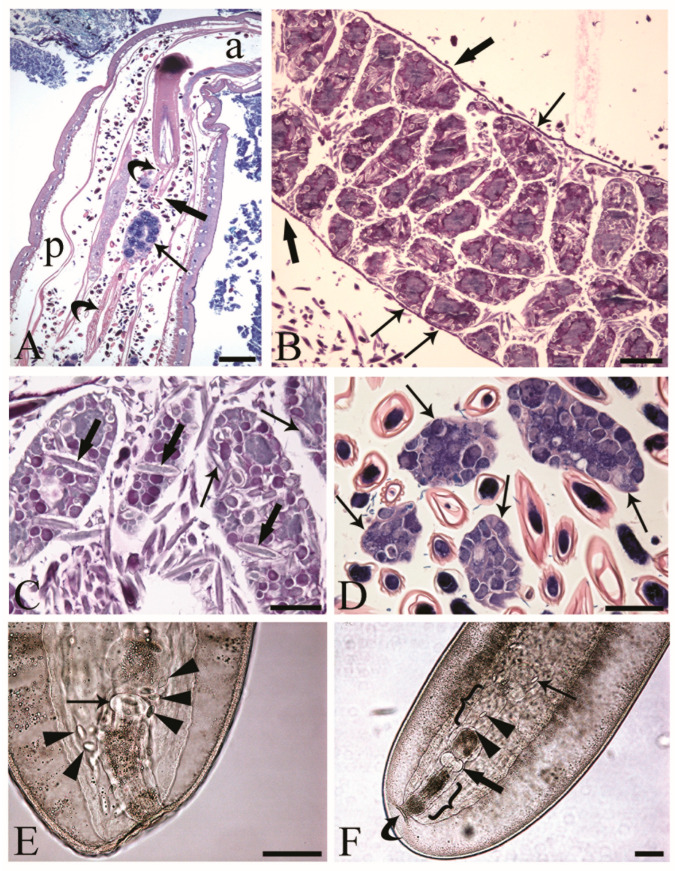
Inseminated female acanthocephalans of *Centrorhynchus globocaudatus.* (**A**) Longitudinal section through fully mature female body; free ovarian ball (arrow) encircled by numerous eggs. Note extension of the ligament sac (curved arrows) from the base of the receptacle in anterior part of body (a) to the posterior end (p) of female body and point of its disruption (thick arrow). Giemsa staining; scale bar, 200 µm. (**B**) Immature female; numerous ovarian balls are still within the lining of the ligament sac (thick arrows) and some are loosely attached to it (arrows). Alcian Blue/PAS staining; scale bar, 100 µm. (**C**) Free ovarian balls in a fully mature female trunk; some eggs including acanthors (arrows) are in the periphery and few (thick arrows) in the inner region of the ovarian balls. Alcian Blue/PAS staining; scale bar, 50 µm. (**D**) Free ovarian balls (arrows) are surrounded by numerous shelled developmental stages. Giemsa staining; scale bar, 50 µm. (**E**) Mounted female *C. globocaudatus*; transition of uterine bell into tube-like uterus (arrow). The organ is surrounded by several shelled acanthors (arrowheads). Scale bar, 100 µm. (**F**) Posterior end of mounted female. A shelled acanthor (thin arrow) enters the inner opening of uterine bell; two acanthors (arrowheads) inside the uterine bell. Uterus (thick arrow) and genital opening (curved arrow) are visible. All the reproductive organs are surrounded by their envelopes inside the genital sheath (brackets); scale bar, 100 µm.

The reproductive tracts of sexually mature *C. globocaudatus* females were differentiated by the ovarian balls and eggs in different stages of development (Figure 1A–C). Primordial gonadal tissue gives rise to multiple separate ovaries, which in turn decompose into even more ovarian balls, and the process continues until large numbers have been formed (Figure 1B). The ovarian balls of a mature female have no permanent attachment to other tissues of the worm; they are contained in the fluid of the body cavity, either freely (Figure 1A,C,D) or loosely constrained in a ligament sac (Figure 1B). In the body of the female *C. globocaudatus*, the ligament sac extends longitudinally through the body, starting from the outer (posterior) surface of the base of the receptacle (Figure 1A) and ending with the uterine bell (Figure 1F). At the terminal part of the female’s body, the uterine bell connects to the uterus and genital opening—the vagina (Figure 1F). Observations of mounted female specimens in lactophenol allowed us to document the transition of the shelled acanthors from the body cavity through the anterior portion of the uterine bell to the uterus (Figure 1E,F, respectively). In *C. globocaudatus*, the opening of the uterine bell resembles a funnel (Figure 1E); several eggs encircle the organ and some enter the funnel and proceed to move through the uterine bell (Figure 1F). In immature *C. globocaudatus* females, the ligament sac is filled with numerous ovarian balls and eggs (Figure 1B). In further development, the single ligament sac disintegrates and the eggs and ovarian balls become dispersed within the pseudocoelom (Figure 1A,C,D). Moreover, eggs containing acanthors were frequently noted close to the ovarian balls (Figure 1A,C,D).

**Figure 2:**
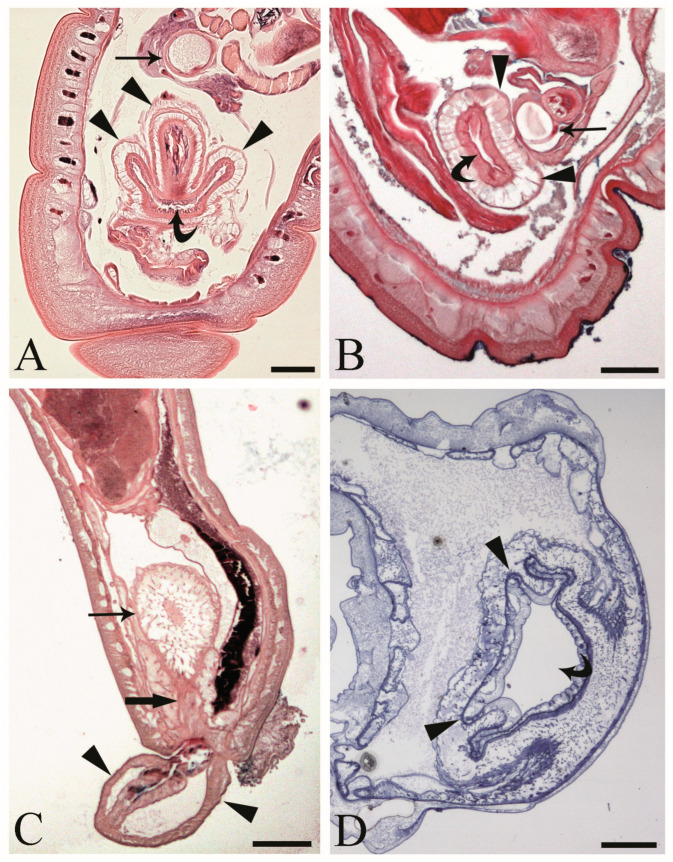
Male acanthocephalan *Centrorhynchus globocaudatus.* (**A**) Longitudinal section of the posterior end shows inverted copulatory bursa and juxtaposition of Saefftigen’s pouch (arrow) and campanulate part of the bursa (arrowheads). Note the opening of the bursa (curved arrow). Alcian Blue/PAS staining; scale bar, 50 µm. (**B**) Close contact between pouch (arrow) and bursa (arrowheads); opening of the bursa is evident (curved arrow). Alcian Blue/PAS staining; scale bar, 50 µm (**C**). Micrograph shows posterior end of male with fully everted bursa (arrowheads), spongy aspect of pouch (arrow), and contact of pouch’s stalk (thick arrow) and campanulate part of bursa. Alcian Blue/PAS staining; scale bar, 200 µm. (**D**) Semi-thin section of terminal part of male body with inverted bursa (arrowheads) and opening of the bursa (curved arrow). Toluidine blue staining; scale bar, 100 µm.

At the hind end of male *C. globocaudatus* specimens, the vesicula seminalis, cement reservoir, Saefftigen’s pouch, and bursa are all close together. When the bursa is inverted, its upper region moves close to the pouch (Figure 2A). In fact, we often observed intimate contact between the pouch and the inverted bursa (Figure 2B). However, the association of the two organs was still obvious when the bursa was everted (Figure 2C). Furthermore, a spongy aspect of the pouch tissue was evident in all specimens examined (Figure 2A–C). The pouch lumen seemed lucid and appeared to be continuous with the vacuolar spaces in the wall of the bursa through the stalk of the pouch (Figure 2C). The bursa itself presented as an organ, ready to grasp the female hind end (Figure 2C). In semi-thin sections, a circular muscle layer delimiting the extension of the organ was evident (Figure 2A,B,D). All male copulatory organs were found to be enveloped by a genital sheath, which was associated with the ligament sac (Figure 2B,C).

### 3.2. Transmission Electron Microscopy (TEM)

**Figure 3:**
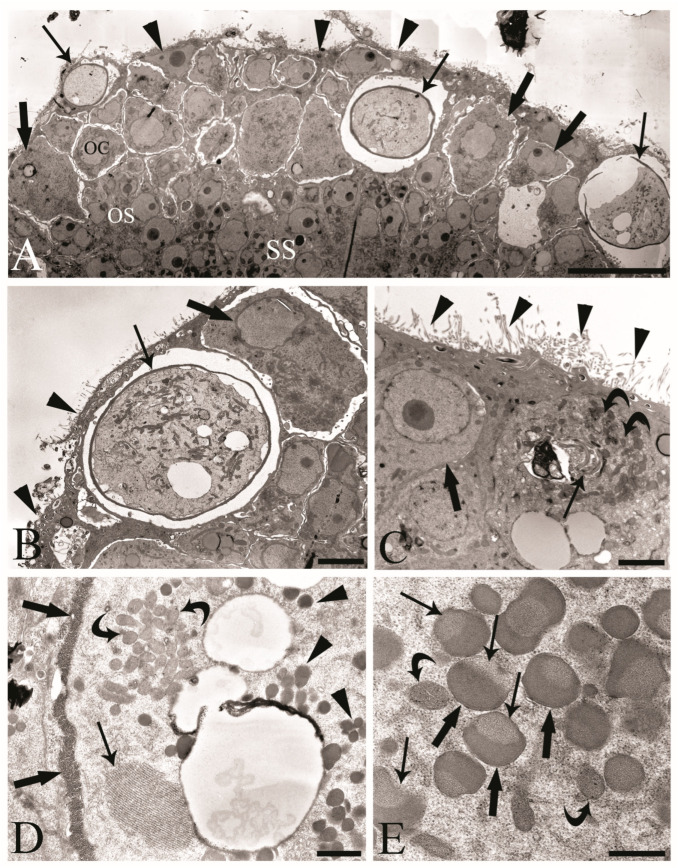
Transmission electron micrographs of the ovarian balls in inseminated female *Centrorhynchus globocaudatus.* (**A**) Section of half of a mature free-floating ovarian ball; internal multinucleate support syncytium (SS), oogonial syncytium (OS), and peripheral germ cell region with oogonial cell (OC), primary oocytes (thick arrows), and inseminated mature oocytes with shells (arrows) are visible. Microvilli (arrowheads) cover the surface of the ovarian ball; scale bar, 1 µm. (**B**) High magnification of periphery of the OB; primary oocyte (thick arrow) with eccentric nucleus, mature oocyte with shell (arrow), and microvilli (arrowheads) cover the surface of the ovarian ball; scale bar, 3.8 µm. (**C**) Micrograph showing the penetration of the spermatozoon into the mature oocyte; sections of the flagellum (arrow) and numerous electron-dense membrane-bound granules (curved arrows) and microvilli (arrowheads) on the surface of the OB; primary oocyte (thick arrow) with eccentric nucleus; scale bar, 2 µm. (**D**) Inseminated mature oocyte with shell in formation (thick arrows); within the cytoplasm, well-developed rough endoplasmic reticulum (arrow) and numerous electron-dense membrane-bound granules (arrowheads) and clusters of mitochondria are visible (curved arrows); scale bar, 0.7 µm. (**E**) In the cytoplasm of the mature oocyte, each membrane-bound granule has an amorphous electron-dense region (thick arrows) and granular region (arrows); curved arrows show mitochondria; scale bar, 0.5 µm.

**Figure 4:**
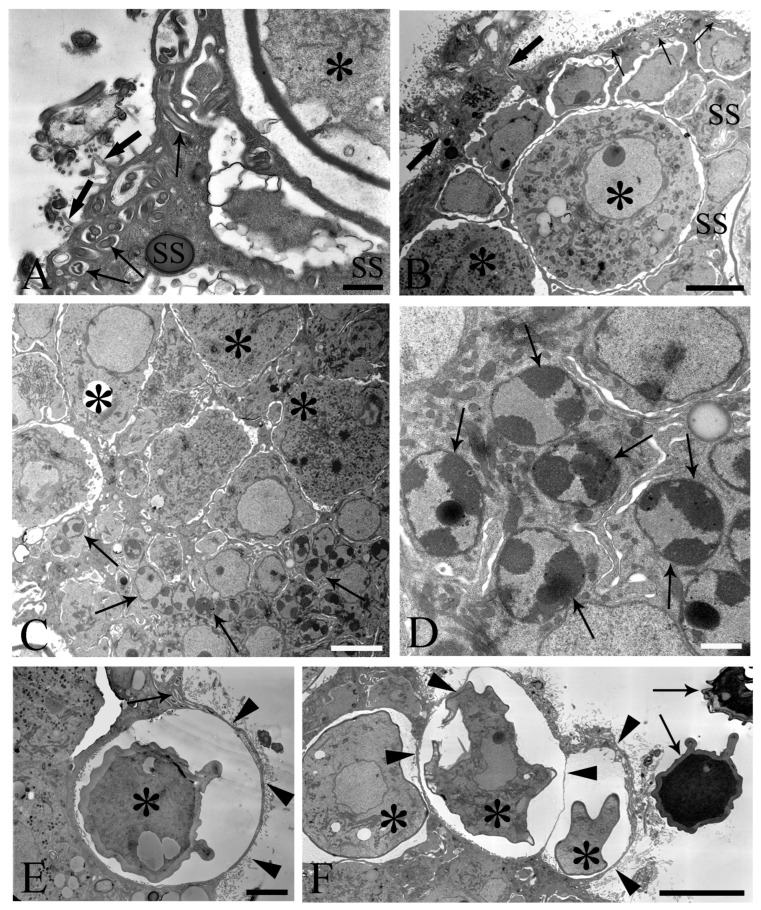
Electron microscope images of ovarian balls in fecundated female *Centrorhynchus globocaudatus.* (**A**) Spermatozoa flagella (arrows) are embedded in the support syncytium (SS); penetration of spermatozoa into ovarian ball via microvillous surface (thick arrows); inseminated mature oocyte (asterisk) with completed shell; scale bar, 0.8 µm. (**B**) Penetration of microvillous OB surface by sperm flagella (thick arrows); flagellum (arrows) embedment in the SS is visible; two inseminated mature oocytes (asterisks) present with numerous electron-dense membrane-bound granules; scale bar, 5 µm. (**C**) Micrograph showing mainly the middle region of an OB with several SS nuclei (arrows) and some primary oocytes (asterisks); scale bar, 5 µm. (**D**) High magnification of the middle region of the OB; aspect of the multinucleate SS is evident; abundant heterochromatin laid on the inner membrane side of some of its nuclei (arrows); scale bar, 1 µm. (**E**) Inseminated mature oocyte (asterisk) with completed shell shortly before disruption of very thin peripheral SS envelope (arrowheads) and release from the OB. Note flagellum (arrow) embedment in SS; scale bar, 3.3 µm. (**F**) Periphery of an OB; some inseminated mature oocytes (asterisks) with shells in formation are peripherally enveloped by a very thin SS layer (arrowheads); after rupturing of the envelope, shelled eggs leave the organ; two free acanthors (arrows) near the OB; scale bar, 3 µm.

We observed the surfaces of the free-floating ovarian balls to be covered with microvilli in female *C. globocaudatus* specimens (Figure 3A–C). These microvilli belonged to the superficial support syncytium. The ovarian ball in the innermost region contained a multinucleate support syncytium (Figure 4C,D), and, toward its periphery, we observed oogonial syncytium, single oogonial cells, and eggs in various developmental stages (Figure 3A). In fact, primary oocytes can co-occur with inseminated oocytes and shelled acanthors close to the support syncytium (Figure 3A,B). Each germ cell is surrounded by its plasmalemma and often two germ cells are close to each other (Figure 4B). In the multinucleate support syncytium (Figure 4C), in each nucleus, heterochromatin was particularly abundant at the inner surface of the nuclear envelope (Figure 4D); indeed, each nucleus was close to the well-developed rough endoplasmic reticulum and not far from several round-elongated mitochondria (). In the primary oocyte, the nucleus has an eccentric position (Figure 3C) and the cytoplasm contains a cluster of mitochondria and a high number of membrane-bound granules (Figure 3D), each granule having an amorphous electron-dense and a clear granular region (Figure 3E). Inside mature oocytes, a well-developed rough endoplasmic reticulum and shells in formation (Figure 3C,D) or fully formed were visible (Figure 3A,B). In Figure 4E, it seems that the inseminated oocytes line up to leave the ovarian ball. We frequently observed free eggs with acanthors enclosed near to the ovarian balls (Figure 1C,D and Figure 4F). It seems that as the oogonia grow in size and become primary oocytes, some move to the periphery of the ovarian ball (Figure 3B) and proceed with differentiation into mature oocytes. The mature oocytes continue to grow; then, when they are fertilized (Figure 3C), they proceed with shell formation (Figure 3D). Afterwards, they release from the ovarian ball surface after rupturing the very thin support syncytium layer (Figure 4E,F). In turn, the spermatozoon first approaches the support syncytium in the peripheral ovarian ball, upon which the sperm plasmalemma moves close to the microvillous surface of the ovarian ball (Figure 4A,B). This process allows the sperm to penetrate deeply, upon which flagellar sections appear in more central areas of the ovarian ball.

**Figure 5:**
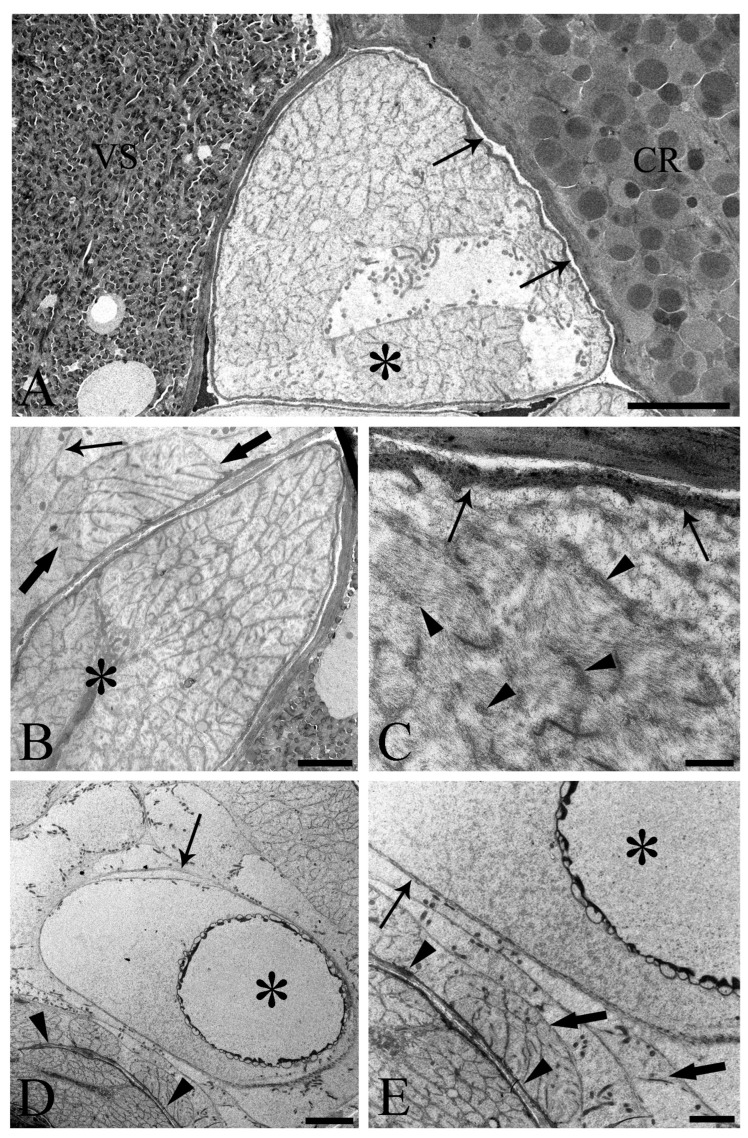
Transmission electron micrographs of sections of mature male *Centrorhynchus globocaudatus* posterior ends. (**A**) Contact between Saefftigen’s pouch (asterisk), vesicula seminalis (VS), and cement reservoir (CR). Each organ has its own lining; often, there is only a narrow distance between these organs (arrows); scale bar, 5 µm. (**B**) Micrograph shows most of the spongy Saefftigen’s pouch (asterisk) in vicinity of the copulatory bursa (arrow); in interface region between both organs appears to be tissue of almost identical aspect to the main portion of Saefftigen’s pouch (thick arrows); scale bar, 2 µm. (**C**) High magnification underscores the spongy aspect of Saefftigen’s pouch, filled with some electron-dense matrix (arrowheads); pouch is delimited by its own lining (arrows); scale bar, 0.5 µm. (**D**) Posterior end of male with portion of the pouch (arrowheads) in proximity to the inverted bursa (arrow); opening of the bursa (asterisk); scale bar, 5 µm. (**E**) Higher magnification of (**D**): in interface region between pouch (arrowhead) and bursa (arrow), occurrence of genital sheath (thick arrows) is evident; opening of the bursa (asterisk); scale bar, 2 µm.

One of the main purposes of the current study was to document the fine structure of the Saefftigen’s pouch and its relationship with the vesicula seminalis, cement reservoir (Figure 5A), and bursa (Figure 5B). The pouch, vesicula seminalis, and cement reservoir are enveloped by thin linings (ranging from 200 to 350 nm, number of measurements 60), which are in close apposition one to another. Often, there was only a very narrow distance between the above organs (Figure 5A,B). Figure 5B illustrates the juxtaposition of the spongy pouch tissue and the bursa. Under very high magnification, it was possible to determine that the spongy aspect was due to the mesh-like inner organization of the pouch tissue (Figure 5C). Contact between the pouch and inverted bursa can be seen also in the TEM observations, which also revealed the campanulate shape of the bursa and its opening (Figure 5D,E). Altogether, the pouch and the other copulatory structures are enclosed in a genital sheath (Figure 5D,E).

## 4. Discussion

Acanthocephala is a monophyletic group of obligate endoparasites with more than 1200 species described to date [1]. Herein, we studied the male and female reproductive systems of the bird parasite *C. globocaudatus* (Acanthocephala: Palaeacanthocephala). We particularly focused on the structure and cytology of the acanthocephalan ovarian balls and the uterine bell in females and Saefftigen’s pouch and associated structures in males, due to the limited information available. Scattered light microscopy studies on the histology of the germ-line constituents of the ovarian balls emphasized the nature of the developing oogonial syncytium stages [6,9,10,11], which was subsequently confirmed by the Feulgen reaction applied to the palaeacanthocephalan *Polymorphus minutus* [24]. A more detailed understanding of the ovarian ball emerged from the TEM investigations [12,13,14,17,25]. These substantiated the general organizational principle of the ovarian balls in acanthocephalans. According to this, the oogonial syncytium originates near the deep supporting syncytium and oogonial cells move to the outer region, thus forming a peripheral zone with cells at different stages of development. Germ cells are surrounded by the supporting syncytium, which also forms the microvillous surface of the ovarian ball and might be derived from the ligament sac primordium [12,14]. Sexual cell divisions in the oogonia and oocytes of two acanthocephalan species were described by Robinson [26]. The present results confirmed the above general organization of the ovarian balls (oogonial syncytium, germ-line cells, zygotes, and shelled eggs within a supporting syncytium) in *C. globocaudatus*. In the ovarian balls, mature oocytes were recognized based on abundant electron-dense membrane-bound granules [2,25,27].

Meyer [11] and others [2,12,27,28,29] reported, for representatives of archi-, eo-, and palaeacanthocephalans, that spermatozoa attach to the surfaces of the ovarian balls. However, there was also mention that fertilization might take place in the body cavity upon the detachment of mature oocytes from the ovarian balls [24,30,31]. The present study revealed the attachment of the spermatozoa to the microvillous surfaces of the ovarian balls in *C. globocaudatus*. Similar to previous investigators [32], we noticed the sperm–ovarian ball attachment to be sufficiently strong to withstand the rigors of fixation and processing for light and electron microscopy. Since we were also able to trace the penetration of spermatozoa into the ovarian balls, fertilization very likely takes place inside the ovarian balls. This will primarily occur in the ovarian ball periphery, but fertilization in deeper regions appears possible too, considering that acanthors were also traced there (Figure 1C). In turn, the many eggs freely floating in the body cavity of the female *C. globocaudatus* were not associated with spermatozoa. Collectively, our results substantiate the interpretation of most authors that fertilization happens while the mature oocytes are still inside the ovarian balls (e.g., [11]).

Upon fertilization, the zygote starts developing into the acanthor larva, acquires a multi-layered shell, and is finally released into the body cavity. On their way from the female body cavity, the eggs usually pass remnants of the ruptured ligament sac [5] and then enter an efferent duct system consisting of the uterine bell, uterus, and vagina [16]. The uterine bell is a complex organ and is located at the inner end of the duct system, but there is no general agreement on the function of this organ. The assumption of an egg sorting function has received support, but others doubt this idea, although without offering an alternative explanation [10,11,33]. After a thorough investigation of the histology and anatomy of the uterine bell of *P. minutus*, it was stated that its morphology matched perfectly with an egg sorting function [18,19]. According to this author, the uterine bell allows embryonated eggs to pass while immature eggs are withheld. Such a function was also preferred in a comparative study of the uterine bells in archi-, eo-, and palaeacanthocephalans [16]. Herein, we documented the occurrence of eggs inside the uterine bell and our results support the egg sorting function.

Until recently, only a few hand-drawn schemes were available on another acanthocephalan peculiarity, Saefftigen’s pouch. These were taken from adult males [20,21] and larvae [15]. Micrographs of Saefftigen’s pouch in a male acanthocephalan were only provided a few years ago [34]. Thus, the light and electron microscopy images presented in this paper are among the first showing the tight contact between the pouch and bursa (see Figure 2C). Similar to the body wall, the bursa is equipped with complex musculature [21], enabling a firm grasp of the female hind end with the everted bursa [35]. Clues regarding the involvement of Saefftigen’s pouch in bursa extrusion have emerged before [21,36]. Asaolu [20] additionally stated that the cavity of the pouch is connected with the vacuolar spaces in the wall of the bursa. It was proposed that the pouch could regulate the turgidity of the wall of the copulatory bursa [20]. The spongy medulla of the pouch contains light material, probably a fluid, which might run through the stalk of the pouch from and into the apical campanulate part of the bursa ([35]; current study). Our observations underline the essential role of Saefftigen’s pouch in the eversion of the bursa for copulation, potentially as a hydrostatic device [21,22,23]. Thus, the concerted action of Saefftigen’s pouch and the bursa probably enables the male to grasp the female hind end prior to insemination. Although copulation must occur frequently, it has been observed only rarely in acanthocephalans. A copulating pair of the palaeacanthocephalan *Dollfusentis heteracanthus* was illustrated by Amin and Dailey [37], and the first light microscope photography of pairs of acanthocephalans in copulation was provided by Dezfuli and De Biaggi [38].

## 5. Conclusions

At present, there are many gaps in our knowledge of acanthocephalan reproduction and further investigations are necessary. This study revealed that the ovarian balls in *C. globocaudatatus* consist of an oogonial syncytium, germ cells, zygotes, shelled eggs, and a supporting syncytium, in which the former are embedded. The present investigation additionally revealed that fertilization occurs not only at the periphery of the ovarian ball but also deeply inside, and it was confirmed that egg sorting is the most likely function of the uterine bell. Herein, we document the connection between Saefftigen’s pouch and the bursa and support the statement that the pouch is essential for the extrusion of the copulatory bursa before the female hind end can be grasped and the spermatozoa transferred.

## Data Availability

Data supporting the reported results can be provided by the corresponding author upon reasonable request.

## Supplementary Materials

The following supporting information can be downloaded at: https://www.mdpi.com/article/10.3390/cells13040356/s1, Figure S1: Anatomical drawing of female and male acanthocephalans.

## References

[1] Smales L.R., Schmidt-Rhaesa A. (2015). Acanthocephala. Handbook of Zoology. Gastrotricha, Cycloneuralia, and Gnathifera.

[2] Crompton D.W.T., Whitfield P.J. (1974). Observations on the Functional Organization of the Ovarian Balls of *Moniliformis* and *Polymorphus* (Acanthocephala). Parasitology.

[3] Herlyn H., De Baets K., Huntley J.W. (2021). Thorny-Headed Worms (Acanthocephala): Jaw-Less Members of Jaw-Bearing Worms That Parasitize Jawed Arthropods and Jawed Vertebrates. The Evolution and Fossil Record of Parasitism.

[4] Sayyaf Dezfuli B., Giari L., Schierwater B., DeSalle R. (2022). Acanthocephala. Invertebrate Zoology—A Tree of Life Approach.

[5] Bullock W.L., Schmidt G.D. (1969). Morphological Features as Tools and as Pitfalls in Acanthocephalan Systematics. Problems in Systematics of Parasites.

[6] Crompton D.W.T., Arnold S., Walters D.E. (1976). The Number and Size of Ovarian Balls of *Moniliformis* (Acanthocephala) from Laboratory Rats. Parasitology.

[7] Crompton D.W.T., Whitfield P.J. (1968). The Course of Infection and Egg Production of *Polymorphus minutus* (Acanthocephala) in Domestic Ducks. Parasitology.

[8] Kates K.C. (1944). Some Observations on Experimental Infections of Pigs with the Thorn-Headed Worm, *Macracanthorhynchus hirudinaceus*. Am. J. Vet. Res..

[9] Hamann O. (1891). Die Nemathelminthen: Beiträge zur Kenntnis ihrer Entwicklung Ihres Baues und ihres Lebensgeschichte.

[10] Kaiser J.E. (1893). Die Acanthocephalen und ihre Entwickelung.

[11] Meyer A., Bronn H.G. (1933). Acanthocephala. Bronn’s Klassen und Ordnungen des Tierreichs. 4. Band.

[12] Atkinson K.H.A., Byram J.E. (1976). The Structure of the Ovarian Ball and Oogenesis in *Moniliformis dubius* (Acanthocephala). J. Morphol..

[13] Asaolu S.O., Whitfield P.J., Crompton D.W.T., Maxwell L. (1981). Observations on the Development of the Ovarian Balls of *Moniliformis* (Acanthocephala). Parasitology.

[14] Peura R., Valtonen E.T., Crompton D.W.T. (1986). Ovarian Tissue in Juvenile Palaeacanthocephalans: *Corynosoma semerme, C. strumosum* and *Echinorhynchus gadi*. Parasitology.

[15] Schmidt G., Crompton D.W.T., Nickol B.B. (1985). Development and Life Cycles. Biology of the Acanthocephala.

[16] Herlyn H., Röhrig H. (2003). Ultrastructure and Overall Organization of Ligament Sac, Uterine Bell, Uterus and Vagina in *Paratenuisentis ambiguus* (Acanthocephala, Eoacanthocephala)—The Character Evolution within the Acanthocephala. Acta Zool..

[17] Davydenko T.V., Nikishin V.P. (2023). Features of Tissue Organization in the Female Reproductive System of *Acanthocephalus tenuirostris* (Palaeacanthocephala, Echinorhynchida). Biol. Bull..

[18] Whitfield P.J. (1970). The Egg Sorting Function of the Uterine Bell of *Polymorphus minutus* (Acanthocephala). Parasitology.

[19] Whitfield P.J. (1968). A Histological Description of the Uterine Bell of *Polymorphus minutus* (Acanthocephala). Parasitology.

[20] Asaolu S.O. (1981). Morphology of the Reproductive System of Male *Moniliformis dubius* (Acanthocephala). Parasitology.

[21] Parshad V.R., Crompton D.W.T. (1981). Aspects of Acanthocephalan Reproduction. Advances in Parasitology.

[22] Miller D.M., Dunagan T.T., Crompton D.W.T., Nickol B.B. (1985). Functional Morphology. Biology of the Acanthocephala.

[23] Hyman L.H. (1951). The Invertebrates: Acanthocephala, Aschelminthes, and Entoprocta—The Pseudocoelomate Bilateria (Volume III).

[24] Nicholas W.L., Hynes H.B.N. (1963). The Embriology of *Polymorphus minutus* (Acanthocephala). Proc. Zool. Soc. Lond..

[25] Marchand B., Mattei X. (1980). Fertilization in Acanthocephala. II. Spermatozoon Penetration of Oocyte, Transformation of Gametes and Elaboration of the “Fertilization Membrane”. J. Submicrosc. Cytol..

[26] Robinson E.S. (1965). The Chromosomes of *Moniliformis Dubius* (Acanthocephala). J. Parasitol..

[27] Peura R., Valtonen E.T., Crompton D.W.T. (1982). The Structure of the Mature Oocyte and Zygote of *Corynosoma semerme* (Acanthocephala). Parasitology.

[28] Marchand B., Mattei X. (1976). Présence de Flagelles Spermatiques Dans Les Sphères Ovariennes Des Eoacanthocéphales. J. Ultrastruct. Res..

[29] Parshad V.R., Crompton D.W.T., Nesheim M.C. (1980). The Growth of *Moniliformis* (Acanthocephala) in Rats Fed on Various Monosaccharides and Disaccharides. Proc. R. Soc. Lond. B Biol. Sci..

[30] Van Cleave H.J. (1953). Acanthocephala of North American mammals. Illinois Biological Monographs.

[31] Guraya S.S. (1969). Histochemical Observations on the Developing Acanthocephalan Oocyte. Acta Embryol. Exp. (Palermo).

[32] Marchand B., Mattei X. (1979). La Fécondation Chez Les Acanthocéphales: I. Modifications Ultrastructurales Des Sphères Ovariennes et Des Spermatozoïdes Après Insémination Des Femelles de l’acanthocéphale *Neoechinorhynchus agilis*. J. Ultrastruct. Res..

[33] Yamaguti S. (1963). Acanthocephala. Systema Helminthum.

[34] Sayyaf Dezfuli B., Rubini S., DePasquale J.A., Pironi F. (2020). Ultrastructure of Male *Centrorhynchus globocaudatus* (Acanthocephala) Cement Apparatus and Function of Cement Gland Secretion. J. Helminthol..

[35] Crompton D.W.T., Crompton D.W.T., Nickol B.B. (1985). Reproduction. Biology of the Acanthocephala.

[36] Yamaguti S., Miyata I. (1938). Notes on *Moniliformis dubius*, Meyer, 1933. Livro Jubil. Prof Travassos Rio Jan. Braz..

[37] Amin O.M., Dailey M.D. (1996). Redescription of *Dollfusentis heteracanthus* (Acanthocephala: Illiosentidae) from Bonefish, Alhula Vulpes, in the West Indies. J. Helminthol. Soc. Wash..

[38] Dezfuli B.S., De Biaggi S. (2000). Copulation of *Acanthocephalus anguillae* (Acanthocephala). Parasitol. Res..

