# Supplementary material for: New Insights on the Male and Female Reproductive Organs of Centrorhynchus globocaudatus (Acanthocephala), Intestinal Parasite of Birds of Prey
Source: Cells. 2024 Feb 18;13(4):356. doi: 10.3390/cells13040356 (PMC10886667; doi:10.3390/cells13040356)
Supplement: Supplementary file 1 [file cells-13-00356-s001.zip › cells-2861181-supplementary.pdf]

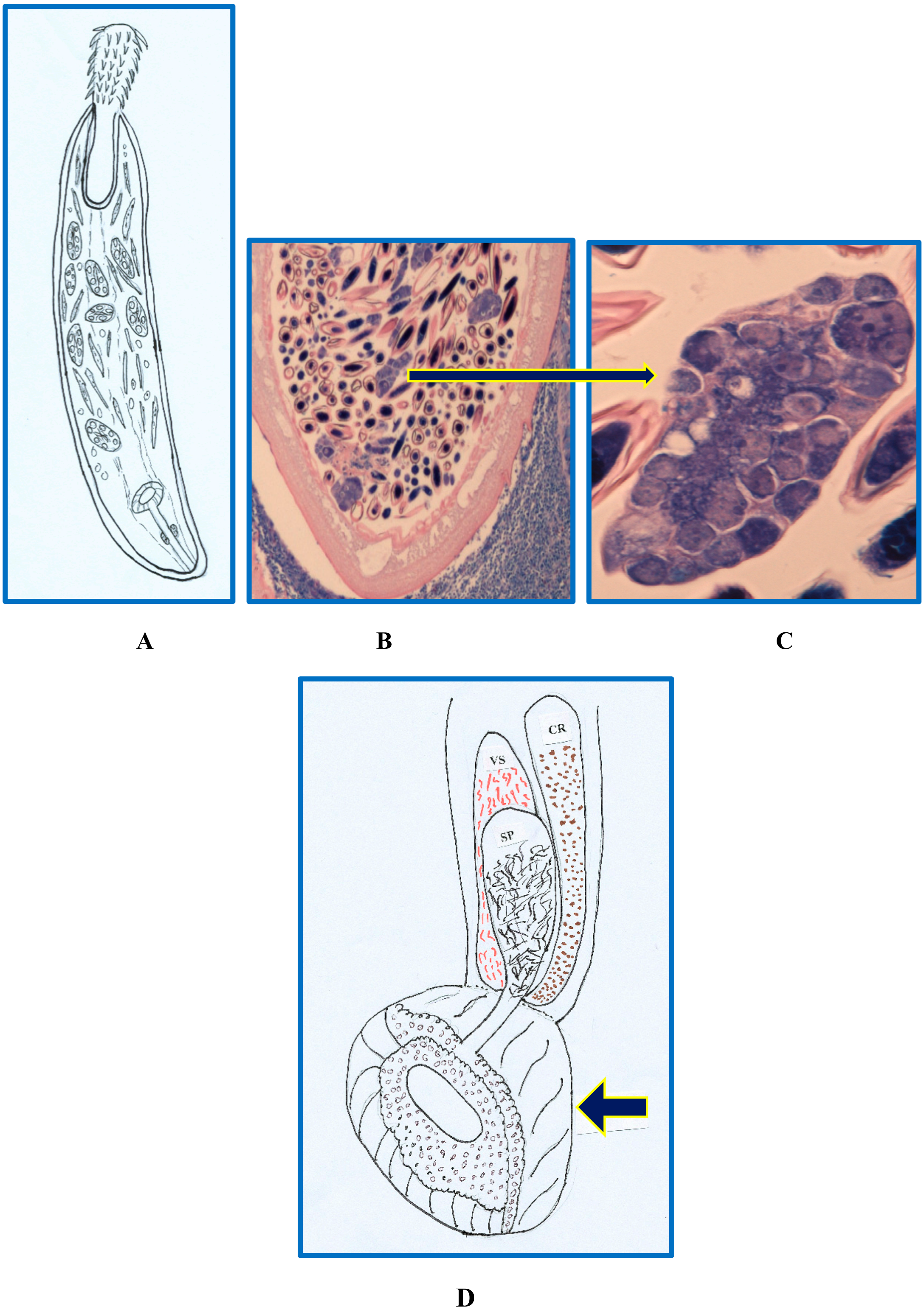

Supplementary Figure S1. (**A**) Anatomical drawing of female acanthocephalan, (**B**) Photo of posterior region of the female, numerous ovarian balls and acanthors are visible, (**C**) High magnification of one ovarian ball. (**D**) Anatomical drawing of posterior region of male acanthocephalan with everted bursa (arrow), SP = Saefftigen’s pouch, VS = Vesicula seminalis, CR = Cement reservoir.
